# Scientific challenges of studying shift schedule design

**DOI:** 10.5271/sjweh.4058

**Published:** 2022-10-01

**Authors:** Dr. Philip Tucker

**Affiliations:** School of Psychology, Faculty of Medicine, Health & Life Science, Swansea University, Swansea, The United Kingdom. Email: p.t.tucker@swansea.ac.uk

The study of shift schedule design, and its impact on the health, safety and the performance of shiftworkers, has seen considerable advances in methodology in the last decade. Much of the early research that helped identify shift schedule design principals was based on observational studies. Quite many featured cross-sectional designs that were subject to residual confounding from unadjusted potential confounders (eg, socioeconomic status, health-driven selection bias). Moreover, the early studies were almost universally based on self-reported exposure and many relied upon self-reported outcomes, making them subject to information bias (1).

Researchers have since built on the findings of earlier research with more sophisticated and robust designs and metrics, many of which are exemplified in studies that have been published in this journal. These provide a firmer evidence base on which to develop recommendations for the design of shift schedules. The fundamental aim of research is to optimise the balance between precision (internal validity), achieved through having control over variables and the study setting; existential realism, achieved through the collection of data in realistic situations; and generalisability of results, from the study sample to the target population (2). In their attempts to achieve this optimal balance, shiftwork researchers have adopted various methodological approaches that try to combine the existential realism (and, in some cases, the generalisability) of the field study with the precision of the laboratory simulation.

The randomised control trial (RCT) is widely regarded as the gold standard of methodological design because confounders are balanced across comparison groups, giving statistical independence between the confounders and the treatment assignment. However, true randomisation to a treatment condition can be impractical, expensive or unethical in field studies that are conducted in the workplace. Hence, true RCT with randomisation at participant level are rare in comparisons of working time arrangements. One of the very few of these types of studies examined the impact of compressed work weeks among police officers who were randomly allocated to either 8-, 10- or 12-hour shifts (3). The results were favourable towards compressing the working week from 8- to 10-hour shifts but somewhat less favourable towards 12-hour shifts.

When the fully experimental approach is not feasible in field settings, another approach to controlling confounders is to use participants as their own control. This approach was adopted in a ‘quasi-experimental’ study (also of police officers), examining the impact of night shift intensity on sleep (4). Each participant undertook three conditions: (i) 2 nights followed by 2 rest days; (ii) 4 nights + 4 rests; and (iii) 7 nights + 7 rests. The order of conditions was allocated individually on a non-random basis that was determined according to the convenience of the rota planner. It was found that sleep was impaired on night shifts and did not improve with an increasing number of consecutive nights, leading to an accumulation of sleep debt with more consecutive nights.

Another form of quasi-experimental study is the group intervention study, where researchers follow the implementation of a change in predictor (eg, working time arrangements) among one group of workers and compare them with a no-change group (a ‘difference-in-difference’ design). Such changes often take place at the instigation of, or are largely guided by, the managers of the organisation in which the study takes place. Thus, potential biases may creep in, such as the way in which groups of participants (eg, departments within the organisation) are allocated to conditions. Moreover, the success of an intervention is likely to be influenced by the process of implementation (eg, the way in which the change is introduced and communicated to the workforce, and the actions of the organisation’s management during implementation). Successful high-quality intervention studies are relatively rare in the study of shift schedule design. Nevertheless, the approach remains a potentially fruitful one, especially in light of the recent publication of a framework for the development of high-quality interventions (5). While the framework’s authors acknowledge the importance of obtaining unbiased estimates (ie, adequately controlling for confounders), they also emphasise the need to understand *how* an intervention contributes to change and the ways in which it interacts with wider dynamic systems within the operational setting.

While intervention studies conducted at the group level remain relatively rare in the study of shift schedule design, recent years have seen an increasing number of observational studies examining the impact of changes in working time arrangements at the individual level. One of the more robust forms of this approach is the non-randomised pseudo trial, in which data from observational studies are analysed in a way that mimics an RCT design (6). This approach was exemplified in a study of night work and the risk of developing common mental disorders (CMD; 7). From baseline, participants were followed up on two occasions, four years apart. At the first follow up, participants were divided into those who had changed schedule since baseline (eg, from non-night to night work) and those who had not, while excluding participants who experienced a change in CMD status in that same initial four-year period (eg, if they had developed a CMD since baseline). At the second follow up, the two groups were compared with respect to their change in CMD status between the first and second follow up. Analyses were also included which adjusted for selection bias, namely the tendency for night workers who develop CMD to transfer to daywork. This bias is a form of the ‘healthy worker effect’ (8), which has long been identified as an obstacle to establishing causal relationships between shift schedule and disease outcomes, and continues to be a challenge to this day.

Case-crossover designs have been employed in several recent studies of shift schedule design. This is essentially a within-participant variation of the case–control study. It involves comparing the relative likelihoods of two scenarios: an outcome event of interest (eg, injury, disease onset or behavioural change) being preceded by the predictor of interest (eg, working a night shift), and the same outcome occurring during a comparable time window when the predictor was not present. The design is most appropriately employed when exposure is intermittent, the effect on risk is immediate and transient, and the outcome is abrupt. Thus, for example, case- crossover designs have been used to study the impact of shift schedule characteristics (eg, shift length, time of day, on-call shifts, shift intensity and weekly work hours) on outcomes such as occupational injuries and on short term sickness absence (9–14). However, the approach has the potential to be applied more widely, eg, to study single changes in exposure level, gradual effects on risk and outcomes with insidious onsets (15).

Turning from study design to measurement, one of the most significant developments in the study of working time arrangements in recent years has been the use of register-based working time data to assess exposure (eg, pay-roll-based registry data of planned and/or executed work hours; 16). The advantage of this approach over traditional self-reported work hours data is that it reduces the risk of information bias due to poor exposure assessment, thereby reducing the risk of exposure misclassification and consequent false negative results. Such precise and accurate exposure assessment is crucial for the identification of etiological relationships, especially when effect sizes are small. Moreover, register-based working time data can provide a fine-grained assessment of exposure over time, which is becoming increasingly important with the trend towards more diverse, flexible and irregular work hours. Some disadvantages of register-based work hours data are that, unlike self-report data, they often only provide exposure assessment over a relatively short historical period (eg, they offer no possibility to assess life-time exposure), and that they only assess exposure within the main job and paid hours (ie, there is usually no assessment relating to second jobs or unpaid work). Moreover, analysing register-based work hours data presents additional challenges for the researcher, compared to traditional data sources. These challenges relate to the processing and cleaning of raw pay-roll data, its transformation into accurate and meaningful schedule parameters, and the substantial computing-power requirements of analysing such very large datasets (M. Härmä – personal communication).

Researchers have also recently drawn upon register data for the assessment of outcomes in relation to working time arrangements. Such studies are most common in the Scandinavian countries where national population-wide register data is collected relating to, for example, the purchase of prescribed medication, hospital treatment, and occupational accidents. Again, register-based assessment eliminates some of the problems commonly associated with self-report (eg, reporting bias and inaccuracy), but it also has potential disadvantages. One potential weakness is that register data is reliant on accurate identification processes eg, accurate diagnosis and consistent treatment decisions by physicians or accurate reporting of accidents at work. This could be especially problematic if the diagnosis- / reporting-behaviour is related to exposure. For example, a physicians’ decisions on diagnosis and treatment may be affected by their patients’ status as a shiftworker or accident reporting procedures at a workplace may differ between day and night time. Some recent studies of working time arrangements have combined register-based assessment of both exposure and outcome, to the exclusion of any self-report measures (eg, 17, 18). While this approach eliminates reporting bias and inaccuracy, the absence of self-report data limits the possibilities for adjusting for potential confounders.

The foregoing only scratches the surface of the diverse range of methodical approaches and measurement techniques available to shift work researchers. Other innovative methods applied to the study of shift schedule characteristics include deriving exposure through job exposure metrics (eg, 19) and through data mining techniques (20), and using co-twin designs to evaluate familial confounding in the impact of nightwork (eg, 21). In addition, increasingly sophisticated wearable technology is available for the intensive measurement of sleep and physiological functioning in field studies (22). Other tools are emerging that may yet prove useful in the study of working time arrangements. For example, today’s researchers studying shift schedule design still often have to rely on observational data and so have much to gain from techniques that strengthen causal inferences made from observational data (23).

While the employer traditionally largely determines shift schedules, there is an increasing trend in some quarters to give shiftworkers a greater voice in their working time arrangements (eg, 24). Irrespective of who is designing the schedules, optimising the design requires balancing factors including productivity, operational logistics, safety, work–life balance and worker health. Science plays an important role in guiding those who are seeking to achieve this balance. The more reliable and accurate the science, the greater its contribution to promoting the health and safety of shiftworkers and the public they serve.
